# Large-scale chemical screen identifies Gallic acid as a geroprotector for human stem cells

**DOI:** 10.1007/s13238-021-00872-5

**Published:** 2021-09-20

**Authors:** Hezhen Shan, Lingling Geng, Xiaoyu Jiang, Moshi Song, Jianxun Wang, Zunpeng Liu, Xiao Zhuo, Zeming Wu, Jianli Hu, Zhejun Ji, Si Wang, Piu Chan, Jing Qu, Weiqi Zhang, Guang-Hui Liu

**Affiliations:** 1 State Key Laboratory of Membrane Biology, Institute of Zoology, Chinese Academy of Sciences, Beijing, 100101 China; 2 State Key Laboratory of Stem Cell and Reproductive Biology, Institute of Zoology, Chinese Academy of Sciences, Beijing, 100101 China; 3 Advanced Innovation Center for Human Brain Protection, National Clinical Research Center for Geriatric Disorders, Xuanwu Hospital Capital Medical University, Beijing, 100053 China; 4 CAS Key Laboratory of Genomic and Precision Medicine, Beijing Institute of Genomics, Chinese Academy of Sciences, Beijing, 100101 China; 5 China National Center for Bioinformation, Beijing, 100101 China; 6 Institute for Stem Cell and Regeneration, Chinese Academy of Sciences, Beijing, 100101 China; 7 University of Chinese Academy of Sciences, Beijing, 100049 China; 8 Aging Translational Medicine Center, Xuanwu Hospital, Capital Medical University, Beijing, 100053 China; 9 Beijing Institute for Stem Cell and Regenerative Medicine, Beijing, 100101 China; 10 Sino-Danish College, University of Chinese Academy of Sciences, Beijing, 101408 China; 11 School of Life Sciences, Beijing University of Chinese Medicine, Beijing, 100029 China

**Dear Editor,**

The interventions that slow aging or promote healthy aging may provide preventative measures for age-related diseases (Zhang et al., 2015). Therefore, it is crucial to identify drugs that target aging-related pathologies and improve healthspan in geroscience research. Using model organisms such as *C*. *elegans* and rodents, several small molecules capable of alleviating the onset or progression of aging, including rapamycin, nicotinamide mononucleotide, and metformin, have been discovered (Partridge et al., 2020). However, the safety and efficacy of these chemicals still need in-depth evaluation before clinical applications (Partridge et al., 2020). As a result, it is necessary to identify additional compounds with geroprotective effects for human cells to counteract the general trend of populational aging. However, transforming a promising compound into an approved drug requires enormous resources. Alternatively, repurposing previously approved drugs for new clinical applications offers a more efficient and less costly path toward drug development. Therefore, testing U.S. Food and Drug Administration (FDA)-approved drugs for geroprotective effects may discover new therapeutics that have already been stringently tested in humans for safety.

Stem cell senescence and exhaustion are key factors of organismal aging. In addition, several cellular hallmarks of aging lead to a decline in stem cell number and function. These hallmarks include genome instability, insufficient self-renewal capacity, heterochromatin erosion, mitochondrial dysfunction, and altered intercellular communication. The human mesenchymal stem cells (hMSCs) are a type of multipotent adult stem cell found in tissue niches across the human body that have been applied for tissue engineering and cell therapy. Several studies have shown that “youthful” factors that rejuvenate senescent hMSCs could mitigate aging-related phenotypes and even extend the lifespan of mammals (Wang et al., 2021). Therefore, identifying compounds that mitigate hallmarks of aging and rejuvenate aged hMSCs is a promising therapeutic strategy to intervene the aging process.

Studying premature aging syndromes (progeria) is crucial for the development of therapies of these conditions and for gaining insight into the process of aging in general. Examples of such syndromes include Werner syndrome (WS) and Hutchinson-Gilford progeria syndrome (HGPS), which primarily affect mesenchymal stem cells. Both WS and HGPS hMSCs display premature aging phenotypes, including insufficient proliferative capacity, severe DNA damage response, and heterochromatin erosion (Wu et al., 2018). WS hMSCs are informative models for phenotypic screening to identify drugs that delay aging due to their accelerated aging kinetics and characteristics of senescence (Zhang et al., 2015; Li et al., 2016). Here, we screened an FDA-approved drug library for compounds that prevent the aging of cells prone to premature aging.

To screen FDA-approved drugs for effects on WS hMSCs (Fig. S1A), we established a high-throughput drug screening platform using the IncuCyte S3 live-cell imaging system (Fig. 1A). A total of 1,622 FDA-approved drugs that possess clinical transforming potential were screened at a concentration of 1 μmol/L for six days in WS hMSCs. Using this preliminary screen, we identified the top 20 candidates that improved the proliferation of WS hMSCs compared to the vehicle control. These candidates include cabozantinib, bicalutamide, plerixafor, azilsartan medoxomil, icotinib, cladribine, baricitinib, alosetron hydrochloride (HCl), minocycline HCl, alverine citrate, gallic acid (GA), rofecoxib, fenoprofen calcium hydrate, amiodarone HCl, doxepin HCl, indacaterol maleate, aztreonam (AZT), lomitapide, deflazacort, and alfacalcidol (Fig. 1B). One of these candidates, baricitinib, is a Janus kinase 1/2 inhibitor that is used to treat rheumatoid arthritis, a prevalent aging-related disorder. In addition, plerixafor is an antagonist of the chemokine receptor CXCR4, rofecoxib and fenoprofen calcium hydrate belong to the nonsteroidal anti-inflammatory drug (NSAID) subgroup, and GA is a natural antioxidant. In sum, we have identified several FDA-approved drugs as potential geroprotectors for hMSCs.

**Figure 1:**
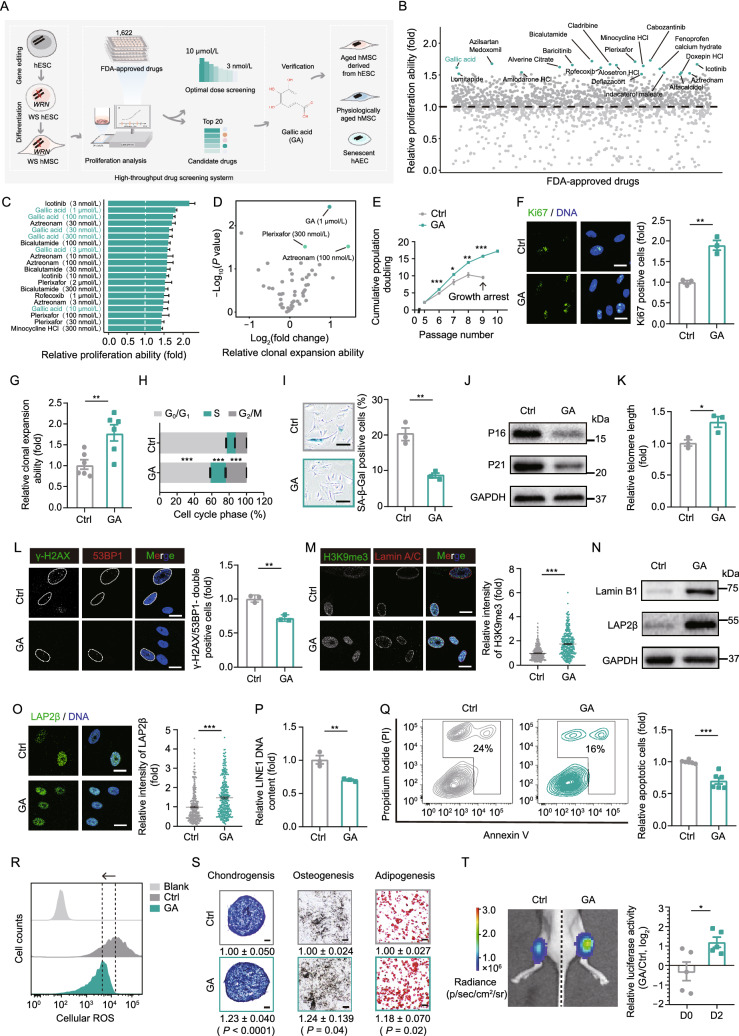
**Large-scale screen in WS hMSCs identifies GA as a potent rejuvenating factor.** (A) Schematic outlining the screening for geroprotective activity of the FDA-approved drug library. (B) The relative proliferation of WS hMSCs (passage 6) treated with various FDA-approved drugs at the concentration of 1 μmol/L. The green dots highlight the top 20 candidate drugs in the primary screening. *n* = 3 biological replicates. The data were normalized to vehicle control (Ctrl). (C) Relative cell proliferation rate upon treatment of WS hMSCs (passage 7) with the top 20 candidate drugs at given concentrations. *n* = 6 biological replicates. The data were normalized to vehicle control. (D) Scatter plot showing the result of clonal expansion assay in WS hMSCs (passage 6) upon treatment of the 18 candidate drugs at the indicated concentrations. The green dots indicate drugs that lead to a significant difference (*P* < 0.05). *n* = 3 biological replicates. (E) Growth curves showing cumulative population doubling of vehicle- and GA-treated WS hMSCs. Data are shown as means ± SEM. *n* = 3 biological replicates. *P < 0.05, **P < 0.01, ***P < 0.001 (*t*-test). (F) Immunostaining of Ki67 in vehicle- and GA-treated WS hMSCs (passage 7), Scale bar, 20 μm. Data are shown as means ± SEM of ≥ 100 cells from three biological replicates. **P < 0.01 (*t*-test). (G) Clonal expansion analysis of vehicle- and GA-treated WS hMSCs (passage 6). Data are shown as means ± SEM. *n* = 6 biological replicates. **P < 0.01 (*t*-test). (H) Cell cycle analysis of vehicle- and GA-treated WS hMSCs by flow cytometry (passage 8). Data are shown as means ± SEM. *n* = 4 biological replicates. ***P < 0.001 (*t*-test). (I) SA-β-Gal staining analysis of vehicle- and GA-treated WS hMSCs (passage 7), Scale bar, 100 μm. Data are shown as means ± SEM of ≥300 cells from three biological replicates. **P < 0.01 (*t*-test). (J) Representative western blot images of P16 and P21 proteins in vehicle- and GA-treated WS hMSCs (passage 8). (K) Quantitative PCR analysis of the relative telomere length of vehicle- and GA-treated WS hMSCs (passage 8). Data represent three independent experiments. Data are shown as means ± SEM. *n* = 3. *P < 0.05 (*t*-test). (L) Immunostaining of γ-H2AX and 53BP1 in vehicle- and GA-treated WS hMSCs (passage 7), Scale bar, 20 μm. White dashed lines represent the nuclear boundaries of cells. Data are shown as means ± SEM of ≥100 cells from three biological replicates. **P < 0.01 (*t*-test). (M) Immunostaining of H3k9me3 and Lamin A/C in vehicle- and GA-treated WS hMSCs (passage 7), Scale bar, 20 μm. Data are shown as means ± SEM of ≥100 cells from three biological replicates. ***P < 0.001 (*t*-test). (N) Representative western blot images of Lamin B1 and LAP2β proteins in vehicle- and GA-treated WS hMSCs (passage 8). (O) Immunostaining of LAP2β in vehicle- and GA-treated WS hMSCs (passage 7), Scale bar, 20 μm. Data are shown as means ± SEM of ≥100 cells from three biological replicates. ***P < 0.001 (*t*-test). (P) Quantitative PCR analysis of the relative LINE1 DNA content in vehicle- and GA-treated WS hMSCs (passage 8). Data represent three independent experiments. Data are presented as means ± SEM. *n* = 3. **P <0.01 (*t*-test). (Q) Analysis of cell apoptosis assay in vehicle- and GA-treated WS hMSCs (passage 8). Data are shown as means ± SEM. *n* = 6 biological replicates. ***P < 0.001 (*t*-test). (R) FACS measurement of ROS levels by staining with the probe H2DCFDA in vehicle- and GA-treated WS hMSCs (passage 8). (S) Characterization of multiple-lineage differentiation potential of vehicle- and GA-treated WS hMSCs (passage 7). Chondrogenic (*n* = 11 spheres), osteogenic (*n* = 3 biological replicates), and adipogenic (*n* = 3 biological replicates) potentials were evaluated by toluidine blue, Von Kossa, and Oil Red O staining, respectively. Scale bar, 100 μm. (T) *In vivo* hMSC implantation assay in nude mice with vehicle (left)- and GA (right)-treated WS hMSCs (passage 8) on Day 0 and Day 2 after implantation. Data calculated by the ratios of log_2_(GA/vehicle) are presented as means ± SEM. *n* = 5 biological replicates. *P < 0.05 (*t*-test)

To further analyze the potential rejuvenating capability of these top candidates, we evaluated the geroprotective effects of the top 20 candidates over a broader range of concentrations: 3 nmol/L, 10 nmol/L, 30 nmol/L, 100 nmol/L, 300 nmol/L, 1 μmol/L, 3 μmol/L, and 10 μmol/L. We found that GA and AZT have positive effects on improving the self-renewal ability of WS hMSCs at multiple concentrations in a 96-well screening system (Fig. 1C). To further verify the geroprotective effect and the optimal dosage of these candidates, we performed clonal expansion assessments on WS hMSCs and found that 1 μmol/L GA is the highest promoting factor of hMSC self-renewal ability (Fig. 1D). The optimum concentration of AZT is 100 nmol/L in the same assay (Fig. 1D). An active and pleiotropic component of black tea and grape seed extract, GA, has been reported to be pharmacologically beneficial as it has anti-inflammatory and antioxidant effects. Therefore, GA was chosen for subsequent analysis.

To investigate the long-term geroprotective effects of GA on WS hMSCs, we treated WS hMSCs with GA for several passages. Compared with vehicle-treated control cells, GA enhanced the self-renewal ability of WS hMSCs as indicated by increased rounds of growth (Fig. 1E). In addition, GA treatment led to increased percentages of Ki67-positive cells (Fig. 1F), improved clonal expansion (Fig. 1G), and increased percentage of cells in the S phase (Fig. 1H) in WS hMSCs. These results suggest that GA has a long-term effect on delaying cellular senescence.

Moreover, GA treatment ameliorated a range of senescent phenotypes in WS hMSCs, including reducing senescence-associated β-galactosidase (SA-β-Gal) positive cells (Fig. 1I). Meanwhile, we observed downregulated expression of the senescent markers P16 and P21 (Figs. 1J and S1B), extended telomere length (Fig. 1K), and the suppression of the DNA damage response (DDR) (Fig. 1L). Interestingly, GA treatment also increased histone H3 Lys9 trimethylation (H3K9me3) in WS hMSCs (Fig. 1M). This covalent histone modification is essential for heterochromatin maintenance and decreased in physiologically and prematurely aged hMSCs (Zhang et al., 2015). Furthermore, GA also upregulated the expression of nuclear envelope proteins Lamin B1 and LAP2β (Figs. 1N, 1O and S1B), which facilitated the maintenance of heterochromatin architecture. Moreover, epigenetic instability was suppressed by GA treatment (Fig. 1P), manifested as aberrant activation of retrotransposons such as the long interspersed nuclear elements 1 (L1 or LINE1) that stimulates the type I interferon response during aging (De Cecco et al., 2019). The GA treatment also reduced cellular apoptosis and ROS levels in WS hMSCs compared to the vehicle control (Figs. 1Q, 1R and S1C). Next, we evaluated the mitochondrial homeostasis in WS hMSCs since mitochondrial dysfunction was also observed during cellular aging. We found that GA treatment increased mitochondrial membrane potential and decreased mitochondrial mass as well as mitochondrial ROS levels (Fig. S1D–F). These measurements indicate an improved mitochondrial function in GA-treated cells. In conclusion, these results demonstrate that GA enhances genomic and epigenetic stability as well as mitochondrial homeostasis in WS hMSCs. In addition, in line with alleviated hMSC senescence, we found that GA enhanced the differentiation potential of WS hMSCs towards osteoblasts, chondrocytes, and adipocytes (Figs. 1S and S1G). GA also increased the *in vivo* retention of WS hMSCs implanted into the anterior tibialis muscles of nude mice (Fig. 1T). These data collectively indicate that GA is a potent geroprotective factor for WS hMSCs.

Next, we tested whether the geroprotective effects of GA could be extended to other progeria models, such as HGPS hMSCs that carried a heterozygous mutation of *LMNA*^G608G/+^ (Wu et al., 2018) (Figs. 2A and S1H). We found that the level of progerin, the truncated and pathogenic protein product of the mutated *LMNA* gene, was decreased by GA treatment in HGPS hMSCs (Fig. S1I). We observed that GA also rescued the senescence phenotypes of HGPS hMSCs, including improved proliferative ability, decreased ratios of SA-β-Gal positive cells, decreased P16 protein levels, and DNA damage response (Figs. 2B–D and S1J–L). HGPS hMSCs treated with GA maintained a relatively normal nuclear morphology with increased levels of heterochromatin mark, H3K9me3, and the nuclear envelope protein LAP2β (Figs. 2C and S1L–O). Furthermore, we found that GA treatment also rescued senescent phenotypes in replicative-senescent (RS) hMSCs, physiologically aged (PA) hMSCs and senescent wild type (WT) hMSCs induced by various types of stresses including ultraviolet (UV) radiation and H_2_O_2_ treatment (Figs. 2E–G and S2A–L). Besides hMSCs, GA also exhibited geroprotective effects on RS human arterial endothelial cells (hAECs) (Figs. S2M and S2N). These data further suggest that GA exerts geroprotective effects across multiple cell models of aging.

**Figure 2:**
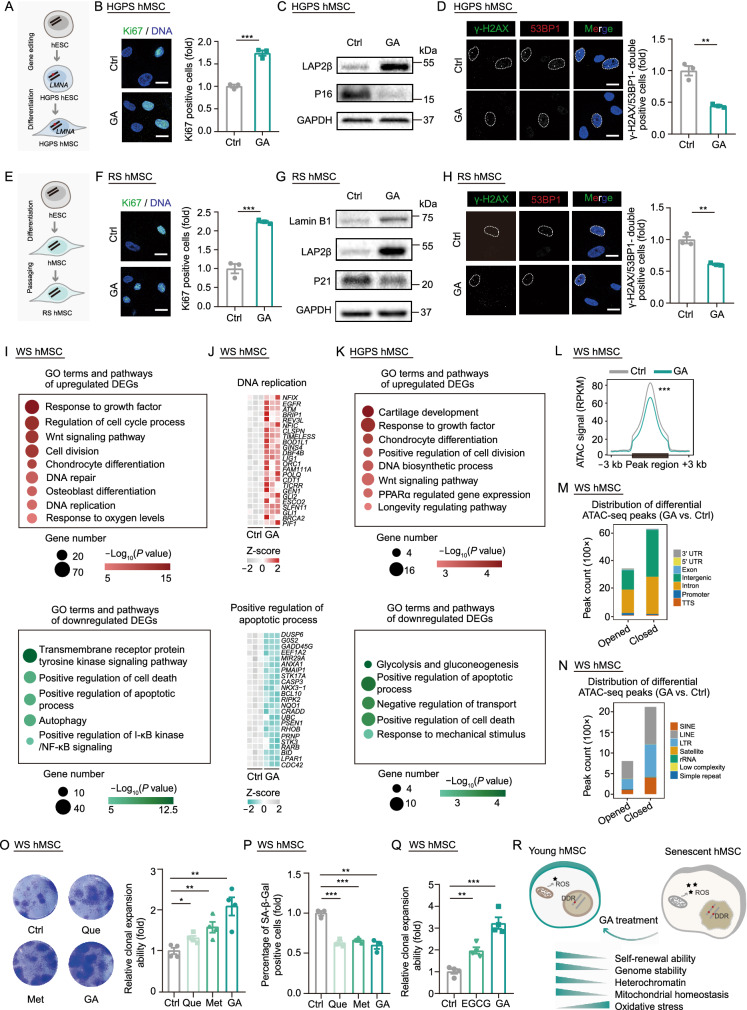
**GA alleviates senescence and enhances self-renewal ability in HGPS, RS and stress-induced senescent WT hMSCs.** (A) Schematic outlining generation of HGPS hMSC. (B) Immunostaining of Ki67 in vehicle- and GA-treated HGPS hMSCs (passage 9), Scale bar, 20 μm. Data are shown as means ± SEM of ≥100 cells from three biological replicates. ***P < 0.001 (*t*-test). (C) Representative western blot images of P16 and LAP2β proteins in vehicle- and GA-treated HGPS hMSCs (passage 9). (D) Immunostaining of γ-H2AX and 53BP1 in vehicle- and GA-treated HGPS hMSCs (passage 9), Scale bar, 20 μm. White dashed lines represent the nuclear boundaries of cells. Data are shown as means ± SEM of ≥100 cells from three biological replicates. **P < 0.01 (*t*-test). (E) Schematic showing generation of replicative-senescent (RS) wild type (WT) hMSCs. (F) Immunostaining of Ki67 in vehicle- and GA-treated RS WT hMSCs (passage 13), Scale bar, 20 μm. Data are shown as means ± SEM of ≥100 cells from three biological replicates. ***P < 0.001 (*t*-test). (G) Representative western blot images of P21, LAP2β and Lamin B1 proteins in vehicle- and GA-treated RS WT hMSCs (passage 14). (H) Immunostaining of γ-H2AX and 53BP1 in vehicle- and GA-treated RS WT hMSCs (passage 13), Scale bar, 20 μm. White dashed lines represent the nuclear boundaries of cells. Data are shown as means ± SEM of ≥100 cells from three biological replicates. **P < 0.01 (*t*-test). (I) GO terms and pathways enrichment analysis of differentially expressed genes (DEGs) in GA-treated WS hMSCs compared to vehicle (passage 9). (J) Heatmaps showing transcriptional levels of genes enriched in each representative GO term or pathway of GA-treated WS hMSCs compared to vehicle (passage 9). (K) GO terms and pathways enrichment analysis of differentially expressed genes (DEGs) in GA-treated HGPS hMSCs compared to vehicle (passage 8). (L) Metaplot showing the decreased ATAC signals at differential ATAC-seq peaks in GA-treated WS hMSCs compared to vehicle-treated WS hMSCs (passage 9). ***P < 0.001 (Wilcoxon test). (M) Genomic element enrichment analysis of opened and closed ATAC-seq peaks in GA-treated WS hMSCs compared to vehicle-treated WS hMSCs (passage 9). (N) Repetitive sequence element enrichment analysis of opened and closed ATAC-seq peaks in GA-treated WS hMSCs compared to vehicle-treated WS hMSCs (passage 9). (O) Clonal expansion ability of WS hMSCs (passage 6) treated with vehicle, Quercetin (Que), Metformin (Met), or GA for 12 days. Data are shown as means ± SEM. *n* = 4 biological replicates. *P < 0.05, **P < 0.01 (*t*-test). (P) SA-β-Gal staining analysis of vehicle-, Que-, Met- and GA-treated WS hMSCs (passage 7). The relative ratio normalized to vehicle control. Data are shown as means ± SEM of ≥300 cells from three biological replicates. **P < 0.01, ***P < 0.001 (*t*-test). (Q) Clonal expansion ability analysis in vehicle-, EGCG- and GA-treated WS hMSCs (passage 6). The relative cell densities of the clones were measured at 15 days after seeding. Data are shown as means ± SEM. *n* = 4 biological replicates. **P < 0.01, ***P < 0.001 (*t*-test). (R) Schematic diagram illustrating the geroprotective effects of GA on hMSCs. Reactive oxygen stress is abbreviated as ROS and DNA damage response is abbreviated as DDR

We performed genome-wide RNA sequencing (RNA-seq) to probe the potential molecular mechanisms by which GA protects WS hMSCs and HGPS hMSCs from senescence. In WS hMSCs, a total of 941 upregulated genes and 417 downregulated genes were obtained in GA-treated WS hMSCs relative to control (|Log_2_ (fold change) | > 0.5, adjust *P* value < 0.05) with high reproducibility of RNA-seq data (Figs. S3A and S3B, Table S4). Differentially expressed genes (DEGs) upregulated by GA treatment include genes enriched in “regulation of cell cycle process’’, “DNA repair’’, “response to oxygen levels”, and “Wnt signaling pathway” (Figs. 2I, 2J, S3C, and S3D). This further supported the notion that GA could restore the self-renewal ability of WS hMSCs. By contrast, downregulated DEGs in GA-treated WS hMSCs were involved in “positive regulation of apoptotic process”, “positive regulation of I-κB kinase/NF-κB signaling”, and “transmembrane receptor protein tyrosine kinase signaling pathway” (Figs. 2I, 2J, S3C, and S3D), which is consistent with the anti-apoptotic and anti-inflammatory effects of GA. Moreover, GA also downregulated the expression of SASP-related genes in WS hMSCs, including *MMP1*, *IL1B*, *IL8*, *SERPINB2*, and *IGFBP2* (Fig. S3E). Furthermore, we identified a total of 212 upregulated genes and 152 downregulated genes in GA-treated HGPS hMSCs (Figs. S4A–C, Table S4). GO term and pathway enrichment analysis revealed that upregulated genes in GA-treated HGPS hMSCs were involved in “positive regulation of cell division” and “PPARα-regulated gene expression” (Figs. 2K and S4D). Next, we investigated whether GA directly modulates features of the cellular aging and found that the expression levels of several aging-associated DEGs annotated by the Aging Atlas (AA) database (Aging Altas, 2021) were changed upon GA treatment with WS hMSCs and HGPS hMSCs (Figs. S3F and S4E). These data demonstrate that GA treatment of hMSCs impacts the regulation of multiple cellular processes, including the cell cycle, chromosome condensation, and DNA repair.

To further explore how the epigenetic state would be affected by GA on a genome-wide scale, we carried out transposase-accessible chromatin sequencing (ATAC-seq) in vehicle- and GA-treated WS hMSCs. The ATAC signals were decreased at intron and intergenic regions, including LINEs, long terminal repeats (LTRs), and short interspersed nuclear elements (SINEs) in GA-treated WS hMSCs compared to those in vehicle-treated WS hMSCs (Figs. 2L–N and S5A–E). Consistently, quantitative PCR analysis revealed lowered relative LINE1 DNA content indicative of less LINE1 mobilization in WS hMSCs upon GA treatment (Fig. 1P). These results indicate that GA facilitates the maintenance of heterochromatin, which at least partially accounts for its geroprotective role.

Finally, we compared the rejuvenating effect of GA with metformin and quercetin, the two classic geroprotective compounds. Strikingly, we found that the effect of GA was slightly better than metformin at 100 μmol/L (Fang et al., 2018) and quercetin at 100 nmol/L (Geng et al., 2019) in promoting the proliferation of WS hMSCs (Figs. 2O and 2P). We also compared GA with epigallocatechin gallate (EGCG) at the same concentration of 1 μmol/L, the latter is a bioactive ingredient of tea polyphenols reported to have antioxidation, anti-inflammation, and anti-cancer effects as well as to promote healthy aging and extend lifespan in worms, flies, and rats (Pallauf et al., 2017). GA-treatment exhibited better geroprotective effects in WS hMSCs than EGCG treatment, as evidenced by improved hMSC self-renewal ability and decreased percentage of SA-β-Gal positive cells (Figs. 2Q, S6A, and S6B). Since EGCG metabolism produces GA *in vivo*, we speculate that the geroprotective effect of tea polyphenols may be partly attributable to GA production. These results attest GA as a new senescence-alleviating drug with clinical promise.

In this study, we performed the first large-scale screening of 1,622 FDA-approved drugs on human stem cell aging models and discovered several compounds that can alleviate senescence phenotypes. In particular, the top hit GA acted as a geroprotective role in various human stem cell models of aging, including WS hMSCs, HGPS hMSCs, RS hMSCs, PA hMSCs, stress (UV or H_2_O_2_)-induced senescent WT hMSCs as well as RS hAECs. These findings demonstrate that GA may have a widespread potential for alleviating phenotypes associated with mesodermal tissue aging. GA treatment attenuated several senescent cellular hallmarks, including defective proliferative capacity, genome instability, mitochondrial homeostasis imbalance, heterochromatin erosion, and oxidative stress (Fig. 2R). Notably, GA had better effects on delaying the aging of WS hMSCs than other geroprotective molecules, including metformin, quercetin, and EGCG, further highlighting the potential of GA for therapeutic applications. This work expands the range of existing agents that protect human stem cells from aging and broadens the discovery path for identifying drugs that alleviate cellular senescence.

Notably, we have established a sensitive, high-throughput screening system for aging-alleviating drugs. Previously, we have used premature aging stem cells to perform low-throughput chemical library screening and successfully identified vitamin C and quercetin as potent geroprotectors to alleviate hMSCs senescence or extend healthspan in mice (Li et al., 2016; Geng et al., 2019). Here, we combined our screening system with automated image acquisition and analysis, and used this high-throughput screening to identify multiple geroprotective candidates, including GA. Among the identified candidates, barictinib has been reported to alleviate senescence features of HGPS patient-derived fibroblasts by inhibiting the JAK-STAT pathway, including the reduction of the expression of progerin and the generation of inflammatory factors (Liu et al., 2019). This further supports the reliability and effectiveness of our high-throughput drug screening system.

GA is a natural phenolic compound abundantly found in tea, fruit, and wine. Several important pharmacological effects of GA have been reported, including antioxidant, anti-inflammatory, and antineoplastic properties. Previous studies showed that GA could enhance the activity of antioxidant enzymes (catalase and glutathione peroxidase) and rejuvenate immune function in D-gal-induced aging-accelerated mice (Guo et al., 2020). GA can also protect healthy subjects and type 2 diabetes patients from DNA oxidation damage (Ferk et al., 2018). Recently, a study revealed that GA supplementation inhibits the progressive decline of rat embryonic fibroblasts and fetal islet cells cultured *in vitro* (Rahimifard et al., 2020). Consistently, our results show that GA attenuated oxidative stress and inhibited SASP in aged human stem cells. Regarding the potential mechanism of GA in reversing these aging hallmarks, RNA-seq analysis revealed that GA-associated beneficial effects may involve enhanced antioxidant response, elevated DNA repair ability, improved cell cycle kinetics, and inhibited NF-κB pro-inflammatory pathway. Our study provided the first evidence linking GA to resisting oxidation stress, improving mitochondrial function, alleviating SASP, and maintaining genomic and epigenetic stability, in the context of human stem cell aging.

Currently established geroprotective drugs mainly function by targeting nutrient-sensing signals, eliminating senescent cells, supplementing endogenous metabolites, and inhibiting SASP (Partridge et al., 2020). The efficacy of GA is attributed to the rejuvenation of senescent hMSCs, indicated by enhanced osteogenesis, chondrogenesis, and adipogenesis. Given that the effect of GA in delaying aging phenotypes is better than, or at least comparable to the existing geroprotective compounds, our results, together with the notion that metformin has been certificated to enter clinical trials for aging intervention (Campisi et al., 2019), provide important data to support GA-based clinical trials in aging intervention.

In summary, we have developed a large-scale drug screening method for aging-alleviating drugs and identified GA as a new geroprotector for human stem cells. This compound shows potent senescence-attenuating effects based on various hMSC aging models and multiple aging defects evaluated *in vitro*. Having confirmed that GA is geroprotective on hMSCs, further investigations are still required, including testing the effect of GA in alleviating various aging-related disorders such as osteoarthritis, atherosclerosis, cognitive decline and determining whether GA prolongs the lifespan of model organisms of progeria and natural aging*.* Nevertheless, our work provides the foundation of exploring the use of GA to improve human healthspan and treat aging-related diseases.

## Supplementary Information

Below is the link to the electronic supplementary material.

Supplementary file1 (PDF 3606 kb)

Supplementary file2 (XLSX 87 kb)

Supplementary file3 (XLSX 540 kb)

Supplementary file4 (XLSX 12 kb)

Supplementary file5 (XLSX 276 kb)
