# Supplementary material for: Large-scale chemical screen identifies Gallic acid as a geroprotector for human stem cells
Source: Protein Cell. 2021 Sep 20;13(7):532–9. doi: 10.1007/s13238-021-00872-5 (PMC9226226; doi:10.1007/s13238-021-00872-5)

Materials and methods

Cell culture

WS (*WRN*$^{-/-}$) human embryonic stem cells (hESCs), HGPS (*LMNA*$^{G608G/+}$) hESCs and WT hESCs (Line H9, from WiCell Research) were differentiated into *WRN*$^{-/-}$ hMSCs, *LMNA*$^{G608G/+}$ hMSCs, and WT hMSCs as previously described (Wu et al., 2018; Zhang et al., 2015). The hESCs were maintained in mitomycin C-inactivated mouse embryonic fibroblasts (MEFs) in hESC culture medium (Cheng et al., 2019) comprised of 80% DMEM/F12 (Gibco), 20% Knockout serum replacement (Gibco), 2 mM GlutaMAX (Gibco), 0.1 mM non-essential amino acids (NEAA, Gibco), 10 ng/mL FGF2 (Joint Protein Central) and 55 μM β-mercaptoethanol (Invitrogen). hESCs were also cultured in Matrigel (BD Biosciences) with mTeSR medium (STEMCELL Technologies, Vancouver). All hESC-derived hMSCs and physiologically aged (PA) hMSCs isolated from gingiva of a 61-year-old individual were cultured in MSC culture medium containing 90% MEM-α with GlutaMAX (Gibco), 10% fetal bovine serum (FBS, Lot 42F1190K, Gibco), 1% penicillin/streptomycin (Gibco), and 1 ng/mL FGF2 (Joint Protein Central). Human arterial endothelial cells (hAECs) were cultured in endothelial cell growth medium (CC-3162, Lonza). No mycoplasma contamination was detected during cell culture.

Differentiation of hMSCs

As previously described (Duan et al., 2015; Fu et al., 2016; Wang et al., 2018a; Wang et al., 2018b; Yan et al., 2019; Zhang et al., 2015), embryoid bodies were cultured with hMSC differentiation medium until confluent MSC-like cells appeared. Then, hESC-derived hMSCs were stained with hMSC-specific markers (CD73, CD90 and CD105) and sorted for CD73, CD90, and CD105 triple- positive cells using fluorescence-activated cell sorting (FACS) (BD FACS Aria IIIu). Differentiation of WS hMSCs into osteoblasts, chondrocytes, and adipocytes followed previously described protocols (Pan et al., 2016; Zhang et al., 2015); Von Kossa (80045.3, GMS), toluidine blue (89640, Sigma) and oil red O staining (O1391, Sigma) were used to identify osteoblasts, chondrocytes and adipocytes, respectively.

FDA-approved drug library screening

The FDA-approved drug library (L1300, Selleck) included 1,622 drugs approved by the FDA, China Food and Drug Administration, National Drug Code, European Medicines Agency, and other drug management organizations or included in pharmacopoeia. The effect was screened in WS hMSCs (passage 6) with compounds at a final concentration of 1 μM. Product numbers, compound names, solvent, and screening results for the FDA-approved drugs library are listed in Table S1. WS hMSCs with a density of 3,000 cells per

well were seeded in gelatin-coated 96-well plates and incubated with various drugs-containing hMSC medium, which was changed every other day for six days. During treatment, cell growth curves were recorded and cell density was evaluated by phase object confluence (percent) using the IncuCyte S3 live-cell imaging system (Essen BioScience, MI USA) (Matin et al., 2019). Cell proliferation was quantified by normalizing cell intensity values (Day 6) of drug treatment wells to the vehicle control wells (n = 3) and ranked.

To optimize the drug intervention concentration, we performed a secondary screen of the top 20 drugs that emerged from our primary screen using a gradient of doses: 3 nM, 10 nM, 30 nM, 100 nM, 300 nM, 1 μM, 3 μM, and 10 μM using the same screening protocol. The compound names and chemical formulae of the top 20 drugs are listed in Table S2.

Clonal expansion assay

The single-cell clonal expansion assay was performed as previously described (Fu et al., 2019). Cells were seeded in 12-well plates with a density of 2,000 cells per well and treated with either vehicle or the drug on the second day. Freshly prepared vehicle or drug-containing hMSC medium was changed every other day for about 12 days. The harvested cells were fixed with 4% paraformaldehyde (PFA) and stained with 10% crystal violet (C0520, Biohao) for 1 hr. The relative cell density was measured using ImageJ software (version: 1.8.0).

Senescence-associated β-galactosidase staining

SA-β-Gal staining was followed as previously described (Ren et al., 2019). In brief, harvested cells were fixed in 2% formaldehyde and 0.2% glutaraldehyde at room temperature for 5 min and then incubated with SA-β-Gal staining solution at 37°C overnight. The SA-β-Gal positive cells were analyzed using ImageJ software (version: 1.8.0).

Western blot analysis

Cells were lysed with 2× SDS buffer (Sigma-Aldrich). Then BCA kit (Thermo Fisher Scientific) was used to measure protein concentration. For western blot detection, 20 μg protein per sample was subjected to SDS-PAGE electrophoresis and transferred onto the PVDF membrane (Millipore). 5% milk (BBI Life Sciences) was used for blocking, the membrane was incubated with specific primary antibodies at 4°C overnight followed by HRP-Conjugated secondary antibodies at room temperature for 1 hr. Protein bands were captured using Image Lab software (ChemiDoc XRS+ system, Bio-Rad) and quantified with ImageJ (version: 1.8.0).

Flow cytometry analysis

For analysis of cell apoptosis levels, Annexin V-FITC/PI apoptosis detection kit (A211-02, Vazyme) was used. For detection of total intracellular ROS, living cells were incubated with CM-H2DCFDA (C6827, Invitrogen) for 30 min at room temperature. For measurement of mitochondrial ROS levels, cells were stained with fluorescent probe MitoSOX (M36008, Invitrogen) for 20 min at 37°C. Cell cycle analysis was conducted using propidium iodide staining for 30 min at 37°C. Mitochondrial membrane potential (MMP) was analyzed using the Cell Meter™ JC-10 mitochondrion membrane potential assay Kit (22801, AAT Bioquest). MMP levels were calculated using specific fluorescence intensities Ex/Em = 490/590 nm (JC-10 aggregate emission) and 490/530 nm (JC-10 monomer emission) (FL590/FL530). Mitochondrial mass was measured by staining cells with 10 μM nonyl acridine orange (NAO, A1372, Invitrogen) for 20 min at room temperature.

The above measurements were performed using the LSR Fortessa cell analyzer (BD), and the data were analyzed with the FlowJo software (TreeStar, Ashland, OR).

Quantitative real-time PCR (RT-qPCR)

For DNA analysis, genomic DNA was extracted with a DNA extraction kit (TIANGEN). Then RT-qPCR was performed with THUNDERBIRD SYBR qPCR mix (Toyobo) in a CFX384 Real-Time PCR system (Bio-Rad). Results were calculated with the ΔΔCq method. RT-qPCR primers are listed in Table S3.

RNA-seq library construction and sequencing

Total mRNA was isolated for RNA-seq using the NEBNext® Poly (A) mRNA Magnetic Isolation Module. To construct sequencing libraries, we used the NEBNext® Ultra™RNA Library Prep Kit for Illumina and then sequenced libraries on Illumina HiSeq X-Ten platforms with paired-end 150-bp sequencing. Quality control and sequencing were achieved by Novo-gene Bioinformatics Technology.

RNA-seq data processing

RNA-seq data were processed on the "Era" petascale supercomputer of the Computer Network Information Center of CAS. Raw data were trimmed using Trim Galore (version 0.5.0) to remove low-quality reads and Illumina reads with adapters. The clean data were mapped to the human reference genome (hg19) using HISAT2 (version 2.0.4) (Kim et al., 2015). Mapped data were determined using HTSeq (version 0.11.0) (Anders et al., 2015). Differentially expressed genes (DEGs) were calculated using the R package DESeq2 (Love et al., 2014) (version 1.26.0) with the cutoff of Benjamini-Hochberg adjust *P* value less than 0.05 and absolute $\log_2$ (fold change) more than 0.5. The DEGs are listed in Table S4. Terms and pathways enrichment analysis was performed using Metascape (http://metascape.org/gp/) (Zhou et al., 2019). The aging-related genes were obtained from

Aging Atlas database (https://bigd.big.ac.cn/aging/index) (Aging Atlas, 2021). Gene set enrichment analysis (GSEA) was performed using the R package clusterProfiler (version 3.14.3) (Yu et al., 2012).

ATAC-seq library preparation and sequencing

The ATAC-seq libraries were prepared according to previous descriptions (Buenrostro et al., 2013; Wang et al., 2019; Wu et al., 2016). Later on, 30,000 cells of vehicle- and GA-treated WS hMSCs at passage 9 were lysed with 50 μL ice-cold lysis buffer (10 mmol/L Tris-HCl (pH 7.4), 10 mmol/L NaCl, 3 mmol/L MgCl2 and 0.5% NP-40) for 10 min. The nuclei were then obtained through centrifugation at 500 *g* for 5 min, and incubated with the Tn5 transposome in the tagmentation buffer at 37 °C for 30 min (TD501, Vazyme Biotech). Subsequently, we purified fragmented DNA with 100 μL (2×) AMPure XP beads (A63882, Beckman Coulter) and amplified with the following PCR conditions: 72 °C for 3 min, 98 °C for 30 sec, thermocycling at 98 °C for 15 sec, 60 °C for 30 sec, and 72 °C for 30 sec for 12 cycles, followed by 72 °C for 5 min. When PCR amplification was completed, libraries were purified and selected by using 0.55× /1.3×AMPure XP beads. Finally, the sequencing of libraries was achieved on Nova platform.

ATAC-seq data processing

The ATAC-seq raw data were trimmed using Trim Galore (version 0.4.4_dev) to remove low-quality reads and reads with adapters. The clean data were mapped with the human reference genome (hg19) using Bowtie2 (version 2.2.9) (Langmead and Salzberg, 2012) software. To prevent the influence of sequencing bias and depth, we merged all the replicates of each sample and randomly selected the same number of reads (45 million) for subsequent analysis. Next, we used Samtools (version 1.9) (Li et al., 2009) to remove the mapped mitochondrial DNA and Y chromosome reads as well as low-quality reads. Duplicate reads were further removed using Picard (version 1.119) software. The black list regions were removed from the above results using Bedtools (version 2.24.0) (Quinlan and Hall, 2010). We extended each read by 250 bp and normalized read counts by RPKM for each 10 bp using the bamCoverage function, then calculated and drawn using computeMatrix, plotHeatmap and plotProfile function in DeepTools (version 3.3.0) (Ramírez et al., 2016). In addition, we used Macs2 (version 2.1.1) to call peak and used the annotatePeaks function of HOMER (version 4.10.3) (Heinz et al., 2010) software to annotate the peak. To identify overlapped peaks in vehicle- and GA-treated WS hMSCs and determine their relative openness, we used Diffbind (version 2.10.0) (FDR < 0.05 and absolute Fold > 1) for analysis.

Immunofluorescence microscopy

For immunofluorescence, harvested cells on coverslips (Thermo Fisher Scientific) were

washed with PBS, fixed in 4% PFA for 30 min, permeabilized with 0.4% TritonX-100 (Sigma) for 30 min, and blocked for 1 hr with 5% donkey serum (Jackson Immuno Research) diluted with PBS. The cells were then incubated with primary antibody at 4°C overnight. Subsequently, the cells were incubated with the corresponding secondary antibody and Hoechst 33342 (Thermo Fisher Scientific) at room temperature for 1 hr. Images were taken using the Leica SP5 confocal microscope.

In vivo stem cell retention analysis

The WS hMSC implantation analysis was performed as previously described (Zhang et al., 2015). 5×10^5 WS hMSCs expressing luciferase were pretreated with vehicle or 1 μM GA for 30 days and injected into the tibialis anterior muscle of male nude mice aged 5-6 weeks. The mice were then injected with D-luciferin solution (Gold-Bio, luck-500) and imaged by the IVIS Spectrum system (PerkinElmer) to detect luciferase activity. Luminescence intensity was analyzed using Living Image® 4.3.

Antibodies

The primary antibodies used for flow cytometry analysis were anti-CD73 (550741, 1:100) and anti-CD90 (555595, 1:200) from BD Biosciences, anti-CD105 (17-1057, 1:100) from eBioscience. The primary antibodies used for Western blot analysis were anti-P21 (2947S, 1:1,000) from Cell Signaling Technology, anti-LAP2β (cat 611000, 1:2,000), and anti-P16 (550834, 1:500) from BD Biosciences, anti-Lamin B1 (Ab16048, 1:1,000), and anti-Werner's syndrome helicase WRN (Ab200, 1:500) from Abcam, anti-GAPDH (sc-365062,1:5000), and anti-Progerin (sc-81611, 1:100) from Santa Cruz Biotechnology. The primary antibodies used for immunofluorescence were anti-Ki67 (ZM-0166, 1:500) from ZSGB-Bio, anti-Lamin A/C (sc-376248, 1:500) from Santa Cruz Biotechnology, anti-LAP2β (cat 611000, 1:500) from BD Biosciences, anti-H3k9me3 (Ab8898,1:500) from Abcam, anti-ɣ-H2AX (05-636, 1:400) from Millipore and anti-53BP1 (A300-273A, 1:600) from Bethyl Laboratories.

Statistical analysis

All data in our study were presented as the means ± SEM. GraphPad Prism 8 software was used to conduct the two tailed Student's *t*-test. P value < 0.05 was considered statistically significant. *, ** and *** indicate $P < 0.05$, $P < 0.01$ and $P < 0.001$, respectively.

Data availability

RNA-seq and ATAC-seq data sets generated in this study have been deposited in Gene Expression Omnibus (GEO) database with accession number GSE181445. RNA-seq data have also been deposited in the Aging Atlas database (https://bigd.big.ac.cn/aging/index) (Aging Atlas, 2021).

Supplemental Figure Legends

Supplemental figure 1. GA exerts geroprotective effects on WS hMSCs and HGPS hMSCs.

(A) Immunoblotting analysis of WRN in WT and WS hMSCs (passage 7). **(B)** Analysis of P16, P21, Lamin B1, and LAP2β protein levels in vehicle- and GA-treated WS hMSCs (passage 8). Data are shown as means ± SEM. n = 3 biological replicates. *$P < 0.05$, **$P < 0.01$, ***$P < 0.001$ (t-test). **(C)** The statistical analysis of relative cellular ROS in vehicle- and GA-treated WS hMSCs (passage 9). Data are shown as means ± SEM. n = 3 biological replicates. ***$P < 0.001$ (t-test). **(D)** Detection of relative mitochondrial membrane potentials (MMPs) with JC-10 in vehicle- and GA-treated WS hMSCs (passage 9). Data are shown as means ± SEM. n = 3 biological replicates. ***$P < 0.001$ (t-test). **(E)** FACS measurement of mitochondrial mass by NAO in vehicle- and GA-treated WS hMSCs (passage 9). Data are shown as means ± SEM. n = 3 biological replicates. ***$P < 0.001$ (t-test). **(F)** FACS measurement of mitochondrial ROS by MitoSOX in vehicle- and GA-treated WS hMSCs (passage 9). Data are shown as means ± SEM. n = 3 biological replicates. *$P < 0.05$ (t-test). **(G)** Quantitative analysis of cross-sectional areas of chondrocyte spheres (lateral section) (n = 11 spheres), Von Kossa-positive areas (n = 3 biological replicates), and Oil Red O-positive areas (n = 3 biological replicates). Data are shown as means ± SEM. *$P < 0.05$, ***$P < 0.001$ (t-test). **(H)** Confirmation of the heterozygous mutation of *LMNA* by DNA sequencing in WT and HGPS hMSCs (passage 7). **(I)** Immunoblotting analysis of Progerin in vehicle- and GA-treated HGPS hMSCs (passage 9). Data are shown as means ± SEM. n = 3 biological replicates. **$P < 0.01$ (t-test). **(J)** Clonal expansion analysis in vehicle- and GA-treated HGPS hMSCs (passage 8). Data are shown as means ± SEM. n = 3 biological replicates. **$P < 0.01$ (t-test). **(K)** Analysis of SA-β-Gal staining in vehicle- and GA-treated HGPS hMSCs (passage 9). Scale bar, 100 μm. Data are shown as means ± SEM of ≥ 300 cells from three biological replicates. **$P < 0.01$ (t-test). **(L)** Analysis of P16 and LAP2β protein levels in vehicle- and GA-treated HGPS hMSCs (passage 9). Data are shown as means ± SEM. n = 3. **$P < 0.01$, ***$P < 0.001$ (t-test). **(M)** Quantification of abnormal nuclei in HGPS hMSCs (passage 9), Scale bar, 20 μm. White dashed lines represent the nuclear boundaries of cells. Data are presented as means ± SEM of ≥ 100 cells from three biological replicates. *$P < 0.05$ (t-test). **(N)** Immunostaining of H3K9me3 in vehicle- and GA-treated HGPS hMSCs (passage 9), Scale bar, 20 μm. Data are shown as means ± SEM of ≥ 100 cells from three biological replicates. ***$P < 0.001$ (t-test). **(O)** Immunostaining of LAP2β in vehicle- and GA-treated HGPS hMSCs (passage 9), Scale bar, 20 μm. Data are shown as means ± SEM of ≥ 100 cells from three biological replicates. ***$P < 0.001$ (t-test).

Supplemental figure 2. GA delays senescence across various human cell models of

aging.

(A) Clonal expansion analysis of vehicle- and GA-treated RS hMSCs (passage 12). Data are shown as means ± SEM. *n* = 3 biological replicates. **P* < 0.05 (*t*-test). **(B)** Analysis of SA-β-Gal staining in vehicle- and GA-treated RS hMSCs (passage 13). Scale bar, 100 μm. Data are shown as means ± SEM of ⩾ 300 cells from three biological replicates. ****P* < 0.001 (*t*-test). **(C)** Analysis of P21, LAP2β, and Lamin B1 protein levels in vehicle- and GA-treated RS hMSCs (passage 14). Data are shown as means ± SEM. *n* = 3 biological replicates. **P* < 0.05, ***P* < 0.01 (*t*-test). **(D)** FACS measurement of ROS levels by staining with the probe H2DCFDA in vehicle- and GA-treated RS hMSCs (passage 13). Representative result of three biological replicates. **(E)** Immunostaining of Ki67 in vehicle- and GA-treated PA hMSCs (passage 4), Scale bar, 20 μm. Data are shown as means ± SEM of ⩾ 100 cells from three biological replicates. **P* < 0.05 *(t*-test*)*. **(F)** Analysis of SA-β-Gal staining in vehicle- and GA-treated PA hMSCs (passage 7). Scale bar, 100 μm. Data are shown as means ± SEM of ⩾ 300 cells from three biological replicates. **P* < 0.05 (*t*-test). **(G)** Analysis of SA-β-Gal staining in vehicle- and GA-treated WT hMSCs (passage 10) after UV irradiation. Scale bar, 100 μm. Data are shown as means ± SEM of ≥ 300 cells from three biological replicates. ***P* < 0.01, ****P* < 0.001 (*t*-test). **(H)** Clonal expansion ability analysis in vehicle- and GA-treated WT hMSCs (passage 9) after treatment with UV irradiation. Data are shown as the means ± SEM. *n* = 3 biological replicates. **P* < 0.05, ****P* < 0.001 (*t*-test). **(I)** Immunostaining of γ-H2AX and 53BP1 in vehicle- and GA-treated WT hMSCs (passage 10) after treatment with UV irradiation, Scale bar, 20 μm. White dashed lines represent the nuclear boundaries of cells. Data are shown as means ± SEM of ≥ 100 cells from three biological replicates. **P* < 0.05, ****P* < 0.001 (*t*-test). **(J)** Analysis of SA-β-Gal staining in vehicle- and GA-treated WT hMSCs (passage 9) after treatment with 50 μM H_2O_2. Scale bar, 100 μm. Data are shown as means ± SEM of ⩾ 300 cells from three biological replicates. **P* < 0.05, ***P* < 0.01 (*t*-test). **(K)** Clonal expansion ability analysis in vehicle- and GA-treated WT hMSCs (passage 9) after treatment with H_2O_2 (50 μM). Data are shown as means ± SEM. *n* = 3 biological replicates. *P* = 0.49, ***P* < 0.01 (*t*-test). **(L)** FACS measurement of cellular ROS by H2DCFDA in vehicle- and GA-treated WT hMSCs (passage 9) after treatment with 50 μM H_2O_2. Representative result of three biological replicates. **(M)** SA-β-Gal staining analysis in vehicle- and GA-treated hAECs (passage 9). Scale bar, 100 μm. Data are shown as means ± SEM of ⩾ 300 cells from three biological replicates. **P* < 0.05 (*t*-test). **(N)** Clonal expansion ability analysis in vehicle- and GA-treated hAECs (passage 9). Data are shown as the means ± SEM. *n* = 3 biological replicates. **P* < 0.05 (*t*-test).

Supplemental figure 3. RNA-seq analysis of vehicle- and GA-treated WS hMSCs.

(A) Heatmap showing the Euclidean distance between RNA-seq replicates of vehicle- and GA-treated WS hMSCs. **(B)** Volcano plot showing the number of upregulated and downregulated genes upon GA treatment of WS hMSCs. **(C)** Heatmaps showing transcriptional levels of genes enriched in each representative GO term or pathway of GA-treated WS hMSCs compared to vehicle (passage 9). **(D)** Gene set enrichment analysis (GSEA) plots showing representative GO terms related to the functions of GA in WS hMSCs. **(E)** Heatmap showing downregulated DEGs associated with SASP upon GA treatment in WS hMSCs. **(F)** Heatmap showing upregulated and downregulated DEGs associated with aging upon GA treatment in WS hMSCs.

Supplemental figure 4. RNA-seq analysis of vehicle- and GA-treated HGPS hMSCs.

(A) The bar plot showing the percentage of C to T transition at 1824 nucleotide of *LMNA* gene in HGPS hMSCs. **(B)** Heatmap showing the Euclidean distance between RNA-seq replicates of vehicle- and GA-treated HGPS hMSCs. **(C)** Volcano plot showing the number of upregulated and downregulated genes upon GA treatment in HGPS hMSCs. **(D)** Heatmaps showing transcriptional levels of genes enriched in each representative GO term or pathway of GA-treated HGPS hMSCs compared to vehicle (passage 8). **(E)** Heatmap showing upregulated and downregulated DEGs associated with aging upon GA treatment in HGPS hMSCs.

Supplemental figure 5. ATAC-seq analysis of vehicle- and GA-treated WS hMSCs.

(A) Heatmap showing the Euclidean distance between ATAC-seq replicates of vehicle- and GA-treated WS hMSCs (passage 9). **(B)** The distribution of ATAC-seq peaks at different genomic regions in vehicle- and GA-treated WS hMSCs (passage 9). **(C)** Scatter plot showing the differential ATAC-seq peaks identified by Diffbind in GA-treated WS hMSCs compared to vehicle-treated WS hMSCs (passage 9). Red and green dots represent the opened and closed ATAC-seq peaks in GA-treated WS hMSCs, respectively. **(D)** ATAC-seq analysis showing the chromatin accessibility of vehicle- and GA-treated WS hMSCs within differentially opened ATAC peaks. ***P < 0.001 (Wilcoxon test). **(E)** Violin plots showing ATAC signals of repetitive sequence regions within differentially opened ATAC peaks in vehicle- and GA-treated WS hMSCs (passage 9). ***P < 0.001 (Wilcoxon test).

Supplemental figure 6. GA exhibits better geroprotective effects than EGCG in WS hMSCs.

(A) Analysis of SA-β-Gal staining in vehicle-, EGCG- and GA-treated WS hMSCs (passage 7). Scale bar, 100 μm. Data are shown as means ± SEM of ≥ 300 cells from three biological replicates. **P < 0.01 (*t*-test). **(B)** Immunostaining of Ki67 in vehicle-, EGCG-, and GA-treated WS hMSCs (passage 7), Scale bar, 20 μm. Data are shown as means ± SEM of ≥ 100 cells from three biological replicates. *P < 0.05, ***P < 0.001 (*t*-test).

Supplemental table legends

Table S1. Screening results of FDA-approved drug library.

Table S2. Top 20 candidates from FDA-approved drug library screening.

Table S3. Primer sequences.

Table S4. Differentially expressed genes in GA-treated WS hMSCs or HGPS hMSCs compared to vehicle control.

Reference

Aging Atlas C (2021). Aging Atlas: a multi-omics database for aging biology. Nucleic acids research *49*, D825-D830.

Anders, S., Pyl, P.T., and Huber, W. (2015). HTSeq--a Python framework to work with high-throughput sequencing data. Bioinformatics (Oxford, England) *31*, 166-169.

Buenrostro, J.D., Giresi, P.G., Zaba, L.C., Chang, H.Y., and Greenleaf, W.J. (2013). Transposition of native chromatin for fast and sensitive epigenomic profiling of open chromatin, DNA-binding proteins and nucleosome position. Nature methods *10*, 1213-1218.

Duan, S., Yuan, G., Liu, X., Ren, R., Li, J., Zhang, W., Wu, J., Xu, X., Fu, L., Li, Y., et al. (2015). PTEN deficiency reprogrammes human neural stem cells towards a glioblastoma stem cell-like phenotype. Nature communications *6*, 10068.

Fu, L., Hu, Y., Song, M., Liu, Z., Zhang, W., Yu, F.X., Wu, J., Wang, S., Izpisua Belmonte, J.C., Chan, P., et al. (2019). Up-regulation of FOXD1 by YAP alleviates senescence and osteoarthritis. PLoS biology *17*, e3000201.

Fu, L., Xu, X., Ren, R., Wu, J., Zhang, W., Yang, J., Ren, X., Wang, S., Zhao, Y., Sun, L., et al. (2016). Modeling xeroderma pigmentosum associated neurological pathologies with patients-derived iPSCs. Protein & cell *7*, 210-221.

Heinz, S., Benner, C., Spann, N., Bertolino, E., Lin, Y.C., Laslo, P., Cheng, J.X., Murre, C., Singh, H., and Glass, C.K. (2010). Simple combinations of lineage-determining transcription factors prime cis-regulatory elements required for macrophage and B cell identities. Molecular cell *38*, 576-589.

Kim, D., Langmead, B., and Salzberg, S.L. (2015). HISAT: a fast spliced aligner with low memory requirements. Nature methods *12*, 357-360.

Langmead, B., and Salzberg, S.L. (2012). Fast gapped-read alignment with Bowtie 2. Nature methods *9*, 357-359.

Li, H., Handsaker, B., Wysoker, A., Fennell, T., Ruan, J., Homer, N., Marth, G., Abecasis, G., and Durbin, R. (2009). The Sequence Alignment/Map format and SAMtools. Bioinformatics (Oxford, England) *25*, 2078-2079.

Love, M.I., Huber, W., and Anders, S. (2014). Moderated estimation of fold change and dispersion for RNA-seq data with DESeq2. Genome biology *15*, 550.

Matin, F., Jeet, V., Srinivasan, S., Cristino, A.S., Panchadsaram, J., Clements, J.A., and Batra, J. (2019). MicroRNA-3162-5p-Mediated Crosstalk between Kallikrein Family Members Including Prostate-Specific Antigen in Prostate Cancer. Clinical chemistry *65*, 771-780.

Pan, H., Guan, D., Liu, X., Li, J., Wang, L., Wu, J., Zhou, J., Zhang, W., Ren, R., Zhang, W., et al. (2016). SIRT6 safeguards human mesenchymal stem cells from oxidative stress by coactivating NRF2. Cell research *26*, 190-205.

Quinlan, A.R., and Hall, I.M. (2010). BEDTools: a flexible suite of utilities for comparing genomic

features. Bioinformatics (Oxford, England) *26*, 841-842.
Ramírez, F., Ryan, D.P., Grüning, B., Bhardwaj, V., Kilpert, F., Richter, A.S., Heyne, S., Dündar, F., and Manke, T. (2016). deepTools2: a next generation web server for deep-sequencing data analysis. Nucleic acids research *44*, W160-165.
Ren, X., Hu, B., Song, M., Ding, Z., Dang, Y., Liu, Z., Zhang, W., Ji, Q., Ren, R., Ding, J., et al. (2019). Maintenance of Nucleolar Homeostasis by CBX4 Alleviates Senescence and Osteoarthritis. Cell reports *26*, 3643-3656 e3647.
Wang, P., Liu, Z., Zhang, X., Li, J., Sun, L., Ju, Z., Li, J., Chan, P., Liu, G.H., Zhang, W., et al. (2018a). CRISPR/Cas9-mediated gene knockout reveals a guardian role of NF-κB/RelA in maintaining the homeostasis of human vascular cells. Protein & cell *9*, 945-965.
Wang, S., Hu, B., Ding, Z., Dang, Y., Wu, J., Li, D., Liu, X., Xiao, B., Zhang, W., Ren, R., et al. (2018b). ATF6 safeguards organelle homeostasis and cellular aging in human mesenchymal stem cells. Cell discovery *4*, 2.
Wang, Y., Lu, T., Sun, G., Zheng, Y., Yang, S., Zhang, H., Hao, S., Liu, Y., Ma, S., Zhang, H., et al. (2019). Targeting of apoptosis gene loci by reprogramming factors leads to selective eradication of leukemia cells. Nature communications *10*, 5594.
Wu, J., Huang, B., Chen, H., Yin, Q., Liu, Y., Xiang, Y., Zhang, B., Liu, B., Wang, Q., Xia, W., et al. (2016). The landscape of accessible chromatin in mammalian preimplantation embryos. Nature *534*, 652-657.
Wu, Z., Zhang, W., Song, M., Wang, W., Wei, G., Li, W., Lei, J., Huang, Y., Sang, Y., Chan, P., et al. (2018). Differential stem cell aging kinetics in Hutchinson-Gilford progeria syndrome and Werner syndrome. Protein & cell *9*, 333-350.
Yan, P., Li, Q., Wang, L., Lu, P., Suzuki, K., Liu, Z., Lei, J., Li, W., He, X., Wang, S., et al. (2019). FOXO3-Engineered Human ESC-Derived Vascular Cells Promote Vascular Protection and Regeneration. Cell stem cell *24*, 447-461 e448.
Yu, G., Wang, L.G., Han, Y., and He, Q.Y. (2012). clusterProfiler: an R package for comparing biological themes among gene clusters. Omics : a journal of integrative biology *16*, 284-287.
Zhang, W., Li, J., Suzuki, K., Qu, J., Wang, P., Zhou, J., Liu, X., Ren, R., Xu, X., Ocampo, A., et al. (2015). Aging stem cells. A Werner syndrome stem cell model unveils heterochromatin alterations as a driver of human aging. Science (New York, N.Y.) *348*, 1160-1163.
Zhou, Y., Zhou, B., Pache, L., Chang, M., Khodabakhshi, A.H., Tanaseichuk, O., Benner, C., and Chanda, S.K. (2019). Metascape provides a biologist-oriented resource for the analysis of systems-level datasets. Nature communications *10*, 1523.

Supplementary figure 1

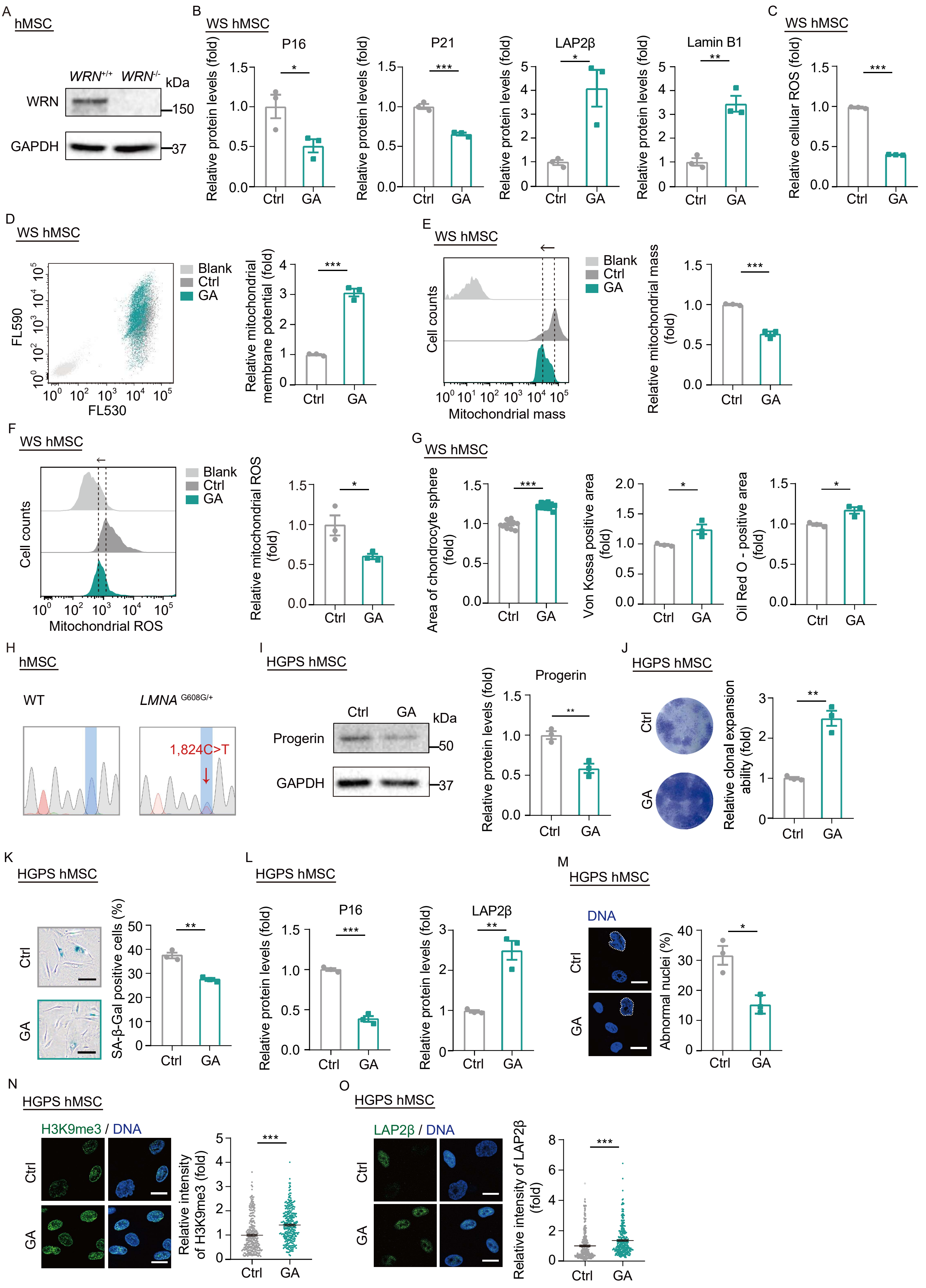

Supplementary figure 2

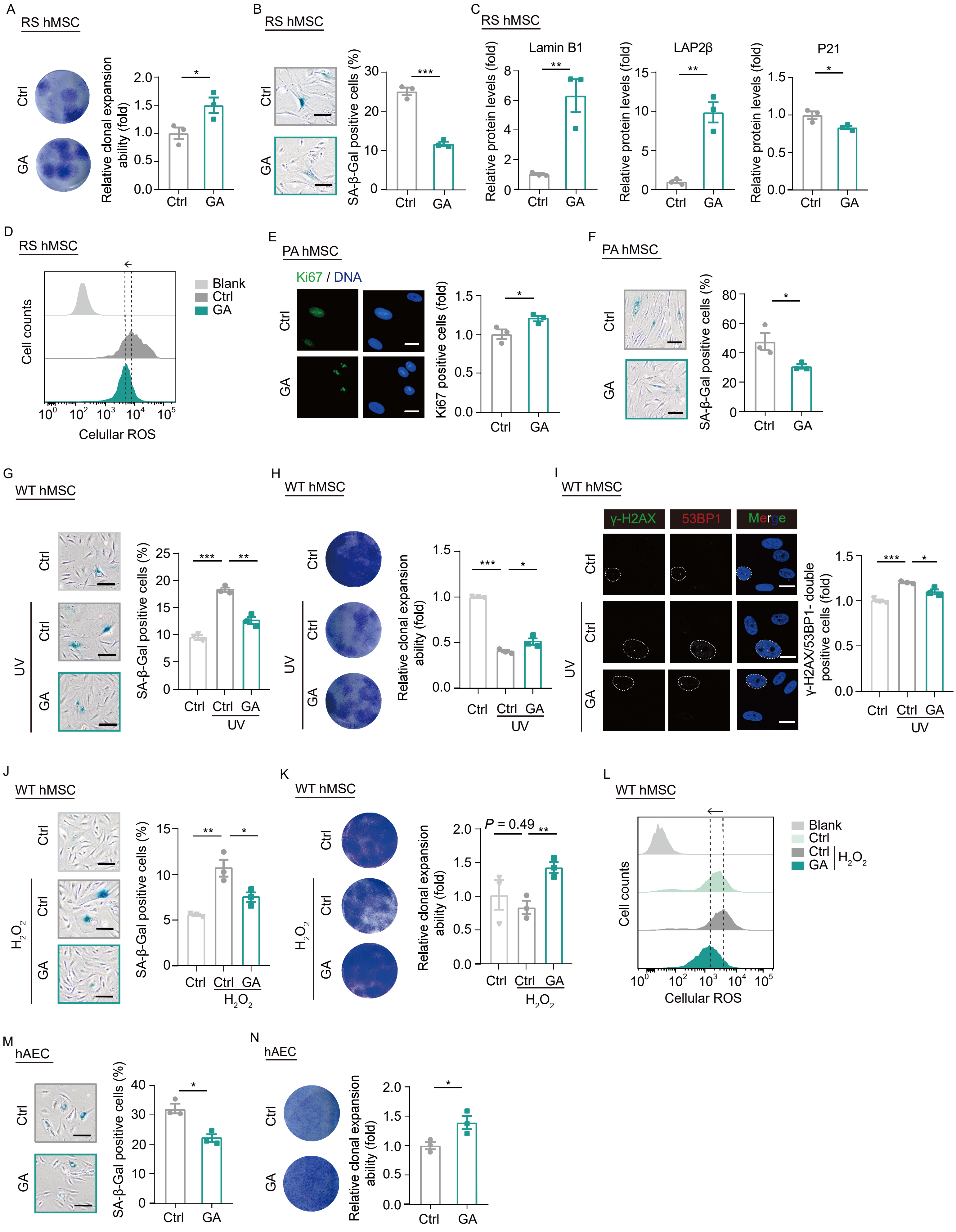

Supplementary figure 3

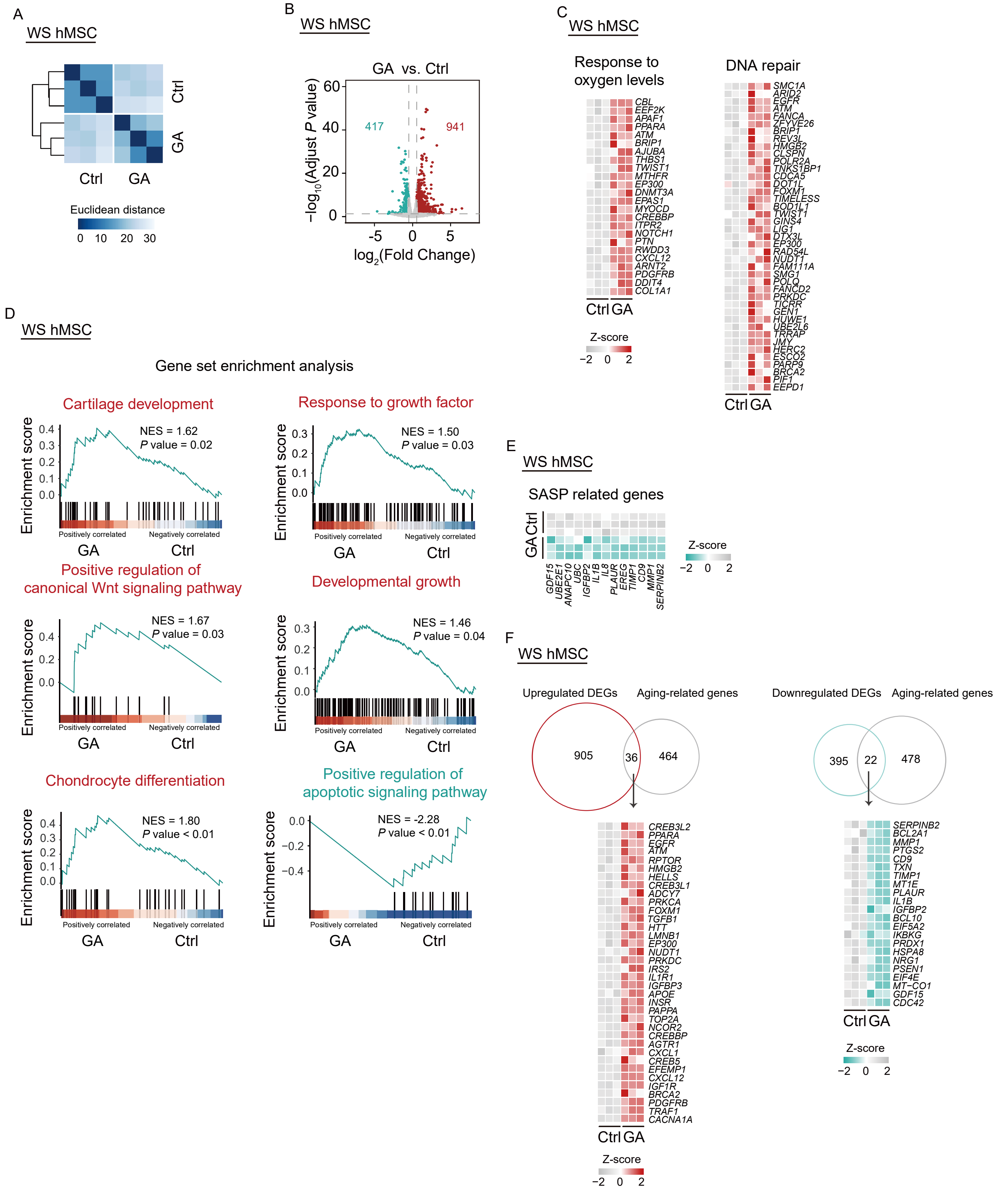

Supplementary figure 4

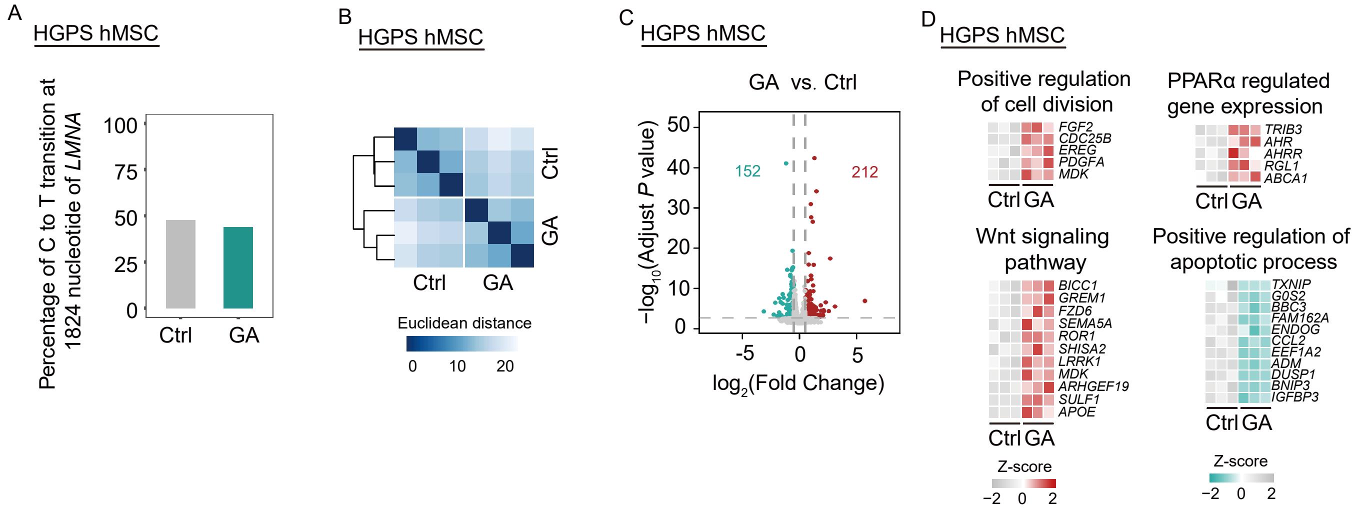

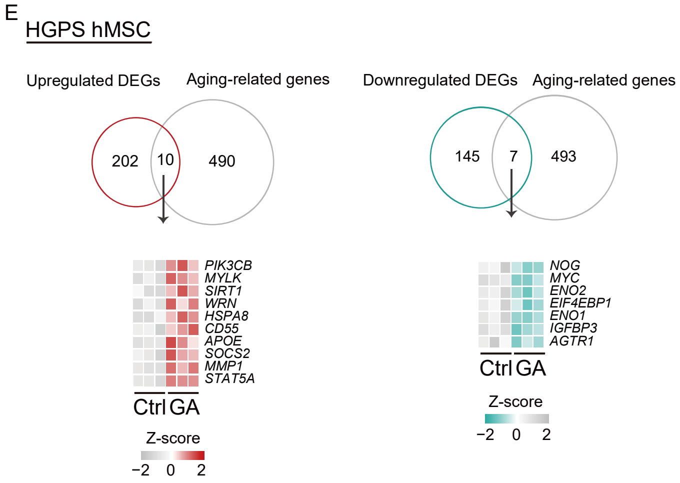

Supplementary figure 5

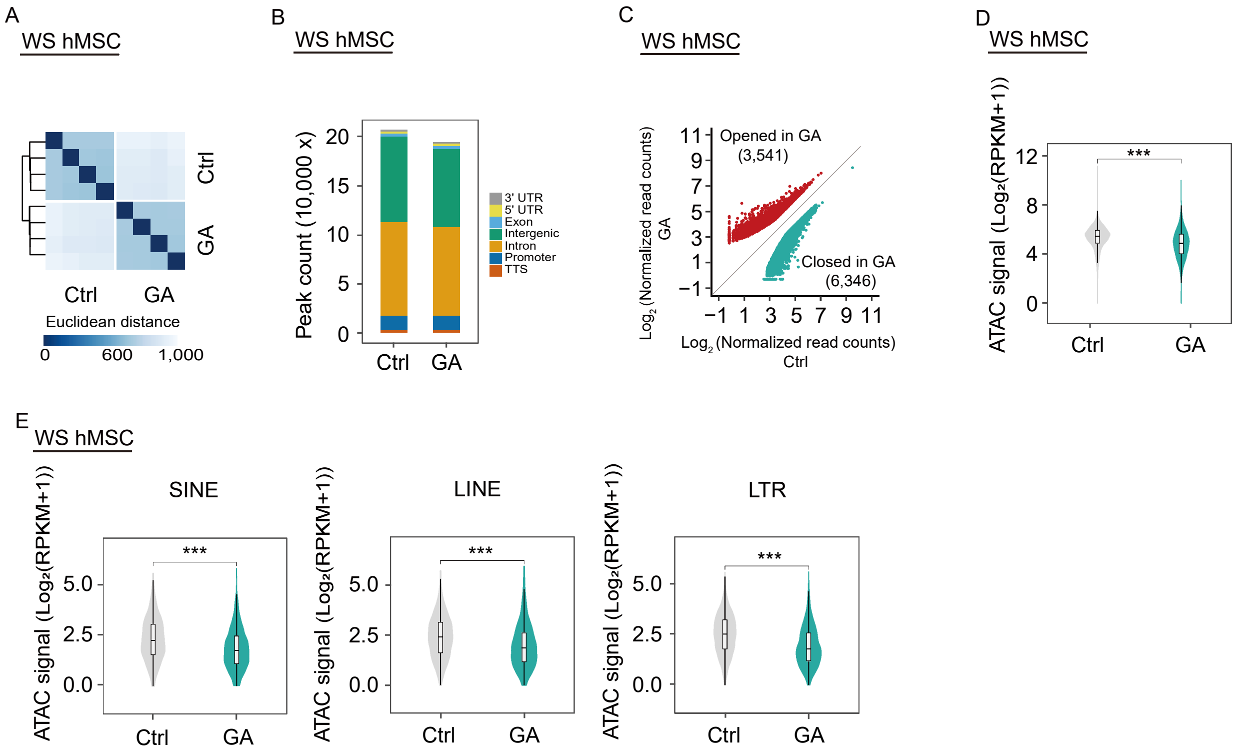

Supplementary figure 6

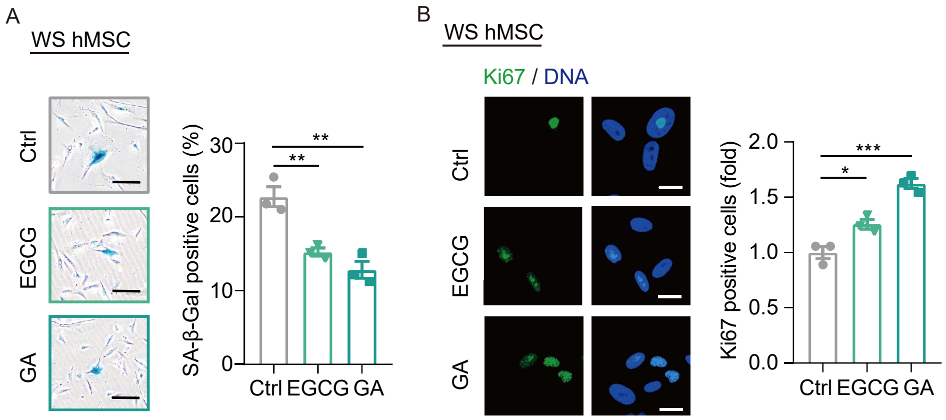

Supplement: Supplementary file 1 — Supplementary file1 (PDF 3606 kb) [file 13238_2021_872_MOESM1_ESM.pdf]
